# Long-term follow-up of patients undergoing renal sympathetic denervation

**DOI:** 10.1007/s00392-022-02056-5

**Published:** 2022-07-18

**Authors:** Victor J. M. Zeijen, Lida Feyz, Rajiv Nannan Panday, Kevin Veen, Jorie Versmissen, Isabella Kardys, Nicolas M. Van Mieghem, Joost Daemen

**Affiliations:** 1 Department of Cardiology, Erasmus University Medical Center, Room Rg-628, P.O. Box 2040, 3000 CA Rotterdam, The Netherlands; 2 Department of Cardiothoracic Surgery, Erasmus University Medical Center, Rotterdam, The Netherlands; 3 Department of Internal Medicine, Erasmus University Medical Center, Rotterdam, The Netherlands

**Keywords:** Antihypertensive agents, Renal artery, Blood pressure monitoring, ambulatory, Hypertension, Kidney, Sympathectomy

## Abstract

**Objectives:**

Renal denervation (RDN) proved to significantly lower blood pressure (BP) at 2–6 months in patients on and off antihypertensive drugs. Given a lack of longer-term follow-up data, our aim was to assess the safety and efficacy of RDN up to five years taking into account antihypertensive drug regimen changes over time.

**Methods:**

In the present single-center study, patients underwent RDN for (therapy resistant) hypertension. Patients underwent protocolized yearly follow-up out to five years. Data were collected on 24-h ambulatory BP and office BP monitoring, renal function, antihypertensive drug regimen, and safety events, including non-invasive renal artery imaging at 6/12 months. Efficacy analyses were performed using linear mixed-effects models.

**Results:**

Seventy-two patients with mean age 63.3 ± 9.5 (SD) years (51% female) were included. Median follow-up time was 3.5 years and Clark’s Completeness Index was 72%. Baseline ambulatory daytime BP was 146.1/83.7 ± 17.4/12.2 mmHg under a mean number of 4.9 ± 2.7 defined daily doses (DDD). At five years, ambulatory daytime systolic BP as calculated from the mixed model was 120.8 (95% CI 114.2–127.5) mmHg and diastolic BP was 73.3 (95% CI 69.4–77.3) mmHg, implying a reduction of -20.9/-8.3 mmHg as compared to baseline estimates (*p* < 0.0001). The number of DDDs remained stable over time (*p* = 0.87). No procedure-related major adverse events resulting in long-term consequences were observed.

**Conclusions:**

The BP-lowering effect of RDN was safely maintained at least five years post-procedure as reflected by a significant decrease in ambulatory daytime BP in the absence of escalating antihypertensive drug therapy over time.

**Graphical abstract:**

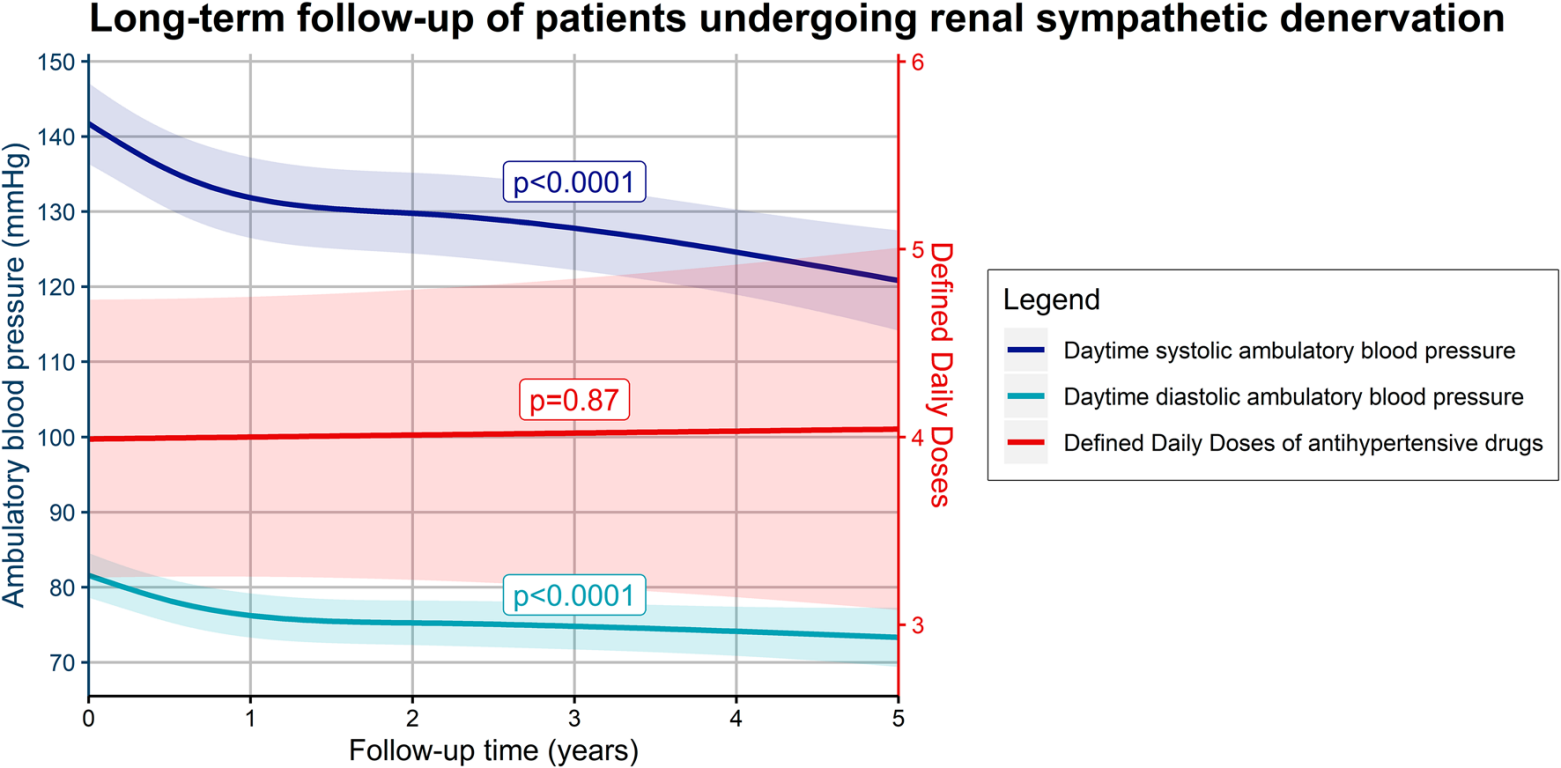

**Supplementary Information:**

The online version contains supplementary material available at 10.1007/s00392-022-02056-5.

## Introduction

Hypertension affects over 1.3 billion people worldwide and poses an increased risk for cardiovascular morbidity and mortality [1–3]. Despite a broad armamentarium of pharmacological and non-pharmacological treatment options for hypertension, most patients do not reach blood pressure (BP) targets proposed in contemporary guidelines [2, 4–7].

By inhibiting afferent and efferent renal sympathetic nerve activity, renal sympathetic denervation (RDN) proved to significantly lower BP in patients on and off antihypertensive medication [8–12]. This resulted in a significantly higher percentage of patients reaching their BP targets following RDN as compared to those post-sham procedures in dedicated randomized sham-controlled trials [8–10].

With the exception of the recently published 3-year data of the sham-controlled SYPRAL HTN-ON MED pilot trial, demonstrating a sustained BP reduction lowering effect of RDN, limited data are available on the long-term safety and efficacy of RDN [13]. While clinical trial data suggested a durable, incremental BP-lowering effect over time, data from animal studies revealed the potential of renal nerve regeneration [14, 15]. Previous single-arm studies refrained from performing long-term ambulatory BP monitoring and lacked detailed information on changes in antihypertensive drug treatment over time complicating the assessment of the durability of the treatment [15].

To address these limitations, the aim of this study was to assess the long-term safety and BP-lowering effect of RDN in hypertensive patients up to five years post-procedure.

## Methods

### Study design and population

This single-center, single-arm registry-based study was conducted at the Erasmus University Medical Center (Rotterdam, the Netherlands). A database consisting of all patients who underwent RDN in our hospital was screened for eligible patients. Patients were included if they underwent RDN for (therapy resistant) hypertension within the scope of a previous clinical study or compassionate use. All patients were aged 18 years or older and used antihypertensive drug(s) prescribed for hypertension or had a documented intolerance to antihypertensive medication.

Participants were informed about the study by the physician responsible for the procedure and provided informed consent for the procedure and the use of anonymous datasets for research purposes in alignment with the Dutch Medical Research Act. This study was conducted in accordance with the declaration of Helsinki.

### Procedure and follow-up

RDN was performed under local anesthesia and conscious sedation according to device-specific instructions for use. Patients were treated with either the endovascular ultrasound (US) ablation Paradise system (ReCor Medical, Inc, Palo Alto, CA, USA) or the radiofrequency (RF) ablation Covidien OneShot™ system (Covidien, Campbell, CA, USA), the RF Vessix system (Boston Scientific, Natick, MA, USA), the RF St. Jude EnligHTN system, the single-electrode RF Symplicity Flex™ system (Medtronic, Inc, Minneapolis, MN, USA) or the multi-electrode RF Symplicity Spyral™ system (Medtronic, Inc, Minneapolis, MN, USA). Following RDN, patients were hospitalized for at least 24 h according to local standard practice. All patients were discharged on aspirin for at least 1 month.

In the Rotterdam RDN clinical program, routine follow-up visits were performed at 3 and 6 months and yearly up to 5 years post-procedure. At each visit, standardized office BP measurement (using automated oscillometric machines), 24-h ambulatory BP measurement and physical examination were performed. Data were also collected on the occurrence of adverse events and renal function (as measured by estimated Glomerular Filtration Rate (eGFR)). While changes in antihypertensive drug regimen were strongly discouraged during the first six months post-treatment, data on time-specific antihypertensive drug regimen were collected at each follow-up visit. Renal artery imaging using either magnetic resonance angiography (MRA) or computed tomography angiography (CTA) was performed in all patients at 6 and/or 12 months of follow-up.

### Endpoints

The primary efficacy endpoint was ambulatory daytime systolic BP (SBP) assessed at multiple points during follow-up. Secondary efficacy endpoints included the use of antihypertensive drugs, ambulatory mean 24-h and nighttime BP, office BP, and office heart rate throughout follow-up. Antihypertensive drug use over time was repeatedly assessed by the number of Defined Daily Doses (DDD), Antihypertensive Load Index (AHLI), and the total number of antihypertensive drugs prescribed [16, 17]. The total number of DDDs per patient was expressed as the sum of the DDDs of each individual prescribed antihypertensive drug. AHLI was calculated according to the formula of Wan et al. as shown below [17]

$$\left(\mathrm{Antihypertensive \,load}=\sum_{antihypertensive \,medications }\frac{\left(prescribed \,daily \,dosage\right)}{\left(maximum \,daily \,dosage\right)}\right).$$

Safety endpoints included the incidence of periprocedural complications, stroke, myocardial infarction (MI), coronary revascularization, hospitalization for hypertensive emergency, newly acquired renal artery stenosis and/or repeat renal artery intervention, renal failure (defined as an eGFR < 15 ml/min/1.73 m^2^ or requirement of dialysis), and all-cause mortality and cardiovascular mortality up to 5 years.

The primary efficacy endpoint was also assessed for pre-specified subgroups of age, sex, ethnicity, obesity, diabetes, isolated systolic hypertension (ISH), and type of RDN device. ISH was defined as office SBP ≥ 140 mmHg and office diastolic BP (DBP) < 90 mmHg at screening [6]. Obesity was defined as a Body Mass Index (BMI) of ≥ 30 kg/m^2^ at screening [18].

Completeness of follow-up was assessed using Clark’s Completeness Index (CCI) which was calculated as the proportion of observed person-years out of the potential maximum number of person-years throughout follow-up [19].

### Statistical analysis

Continuous variables are expressed as mean ± standard deviation (SD) when normally distributed; non-normally distributed variables are presented as median [25th–75th percentile]. Categorical variables are expressed as number of patients (percentage). Continuous repeated measurements were analyzed using linear mixed-effects models. Models were fitted with ambulatory BP, office BP, DDD, AHLI, number of antihypertensive drugs prescribed, or eGFR as outcome variables. In the fixed-effects part, the covariates time, age, and sex were included. In models estimating BP and eGFR outcomes, DDD (repeatedly assessed over time) was also included in the fixed-effects part. In the random-effects part, to account for the presence of multiple measurements per patient, random intercepts and random slopes for time were included. To account for a non-linear effect of time, natural splines with three degrees of freedom were included when appropriate. The appropriate structure best fitting the data was selected using Likelihood Ratio Tests (LRT) and the Akaike Information Criterion (AIC). Statistical hypothesis testing was performed using *F* tests or LRTs and *P* values were reported for the modeled change in the outcome variable over time. Models were visualized using effect plots showing the modeled dependent outcome variable over time including its 95% confidence interval (CI). For models with natural splines, effect estimates and effect plots were given for female patients of mean age taking a mean number of antihypertensive drug DDDs at baseline. For models without natural splines, the regression coefficient for time including its 95% CI was also reported. Exploratory subgroup analyses evaluating a different time effect on the primary outcome in predefined subgroups were performed by testing the significance of adding time–subgroup interaction terms in models estimating the primary outcome (ambulatory daytime SBP). *P* values for the significance of the interaction term were reported for all subgroups, whereas effect plots for subgroups were provided if significant interaction was observed. *P* values < 0.05 were considered statistically significant. All tests were two tailed. Statistical analysis was performed using R version 4.0.2 (Vienna, Austria) with package “nlme” for estimating linear mixed-effects models.

## Results

### Study population

**Fig. 1:**
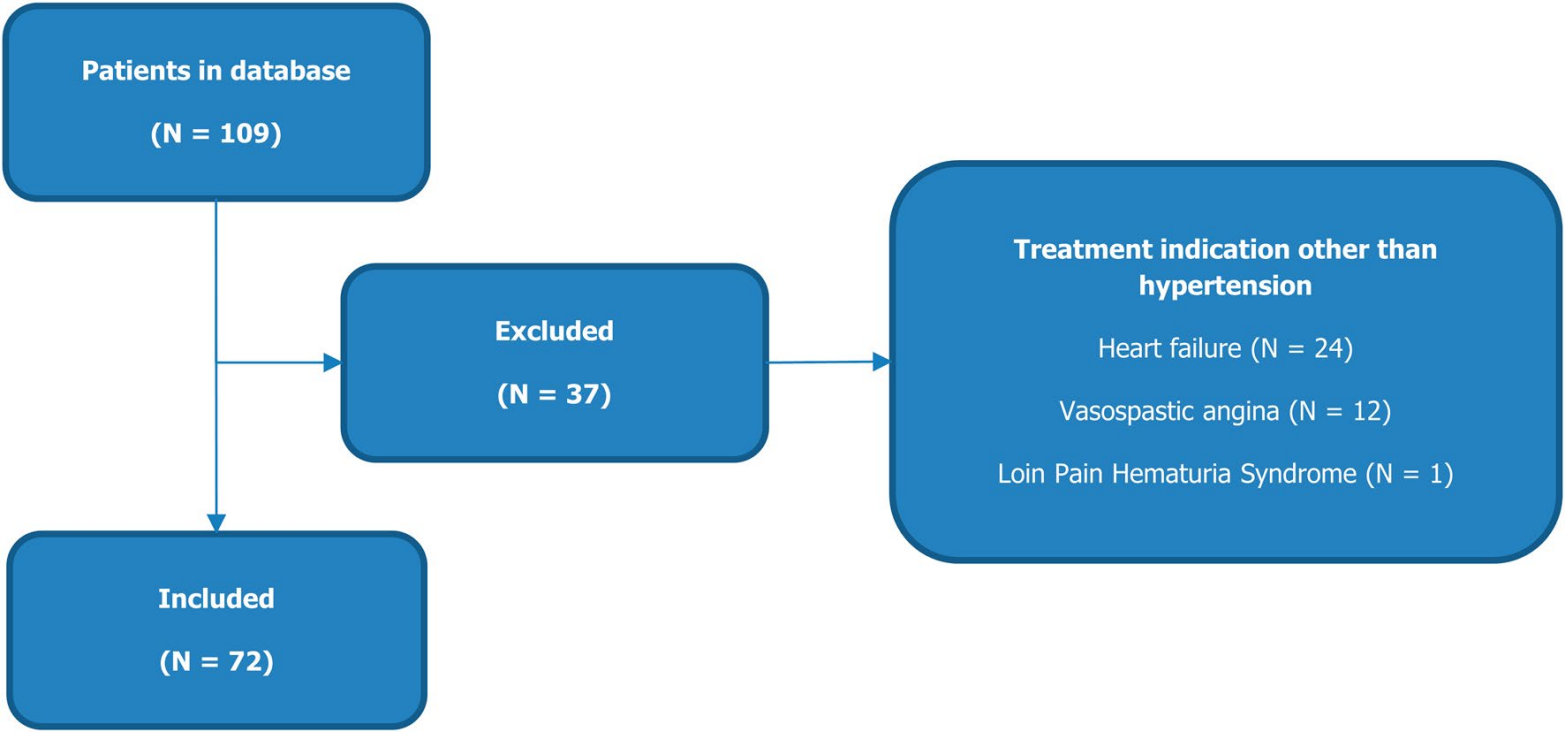
Flowchart for inclusion/exclusion criteria

A total of 109 patients were screened, from which 72 patients, treated between December 2012 and April 2019, met the inclusion criteria. Thirty-seven patients were excluded due to treatment for indications other than hypertension (Fig. 1). Mean (± SD) age at baseline was 63.3 ± 9.5 years and 37 (51.3%) patients were female. Type 2 diabetes was present in 19 (26.4%) of the patients, 27 (37.5%) had ISH, and mean eGFR at baseline was 71.7 ± 16.0 ml/min/1.73 m^2^. Median follow-up time was three and a half years, patients on average performed 6.5 visits, and CCI for follow-up visits was 71.9% (Supplemental Table 1).

At baseline, ambulatory daytime SBP was 146.1 ± 17.4 mmHg and DBP was 83.7 ± 12.2 mmHg. Office SBP was 169.2 ± 21.2 mmHg, whereas DBP was 93.0 ± 14.1 mmHg. Patients were receiving a mean number of 4.9 ± 2.7 DDDs of antihypertensive drugs, while 70 (97.2%) patients were receiving one or more antihypertensive medications at baseline (Table 1).

**Table 1:** Baseline Demographic and Clinical Characteristics of the Study Population

Characteristic	Population (*N* = 72)
Age (years), mean ± SD	63.3 ± 9.5
Female sex, *n* (%)	37 (51.3)
Ethnicity White, *n* (%) Black, *n* (%) Other, *n* (%)	62 (86.1) 8 (11.1) 2 (2.8)
Body mass index (kg/m^2^), median [25th–75th percentile]	29.0 [26.1–32.8]
eGFR (ml/min/1.73m^2^), mean ± SD	71.7 ± 16.0
Diabetes Type 1, *n* (%)	0 (0)
Diabetes Type 2, *n* (%)	19 (26.4)
Electrocardiography	
Sinus rhythm at baseline, *n* (%)	65 (90.3)
Office heart rate prior to procedure (BPM), median [25th–75th percentile]	68.5 [58–8–77.3]
Office blood pressure measurements	
Office SBP (mmHg), mean ± SD	169.2 ± 21.2
Office DBP (mmHg), mean ± SD	93.0 ± 14.1
Isolated Systolic Hypertension, n (%)	27 (37.5)
Ambulatory blood pressure measurements	
Daytime ambulatory SBP (mmHg), mean ± SD	146.1 ± 17.4
Daytime ambulatory DBP (mmHg), mean ± SD	83.7 ± 12.2
24-h ambulatory SBP (mmHg), mean ± SD	143.2 ± 16.4
24-h ambulatory DBP (mmHg), mean ± SD	81.2 ± 11.5
Nighttime ambulatory SBP (mmHg), mean ± SD	134.0 ± 17.7
Nighttime ambulatory DBP (mmHg), mean ± SD	74.8 ± 12.7
Number of antihypertensive medications at screening	
Total number of antihypertensive medications at screening, median [25th–75th percentile]	3.0 [3.0–4.0]
0*	2 (2.8)
1	2 (2.8)
2	9 (12.5)
3	27 (37.5)
4	18 (25.0)
5	10 (13.9)
6	4 (5.6)
Defined Daily Dose (DDD) at screening, mean ± SD	4.9 ± 2.7
Antihypertensive load index at screening, mean ± SD	2.1 ± 1.1
Types of antihypertensive medication at screening within study population	
Angiotensin-converting enzyme inhibitor, *n* (%)	16 (22.2)
Angiotensin receptor blocker, *n* (%)	47 (65.3)
Direct renin inhibitor, *n* (%)	0 (0)
Calcium Channel Blocker, *n* (%)	48 (66.7)
Thiazide Diuretic, *n* (%)	55 (76.4)
Loop Diuretic, *n* (%)	3 (4.2)
Aldosterone antagonist, *n* (%)	10 (13.9)
Alpha-1 receptor blocker, *n* (%)	19 (26.4)
Beta Blocker, *n* (%)	49 (68.1)
Centrally acting agent, *n* (%)	0 (0)

*eGFR* indicates estimated glomerular filtration rate, *SBP* systolic blood pressure, *DBP* diastolic blood pressure

*Two patients were drug intolerant

### Procedural characteristics

Median [25th–75th percentile] procedural time was 60.0 [50.0–75.0] minutes using a contrast volume of 80.5 [61.3–140.0] mL. In five cases (6.9%) a unilateral procedure was performed due to anatomic ineligibility for treating one of the renal arteries (Table 2).

**Table 2:** Procedural characteristics

Procedural characteristics	Treated patients (*N* = 72)	
Procedural time (min), median [25th–75th percentile]	60.0 [50.0–75.0]	
Amount of contrast used (mL), median [25th–75th percentile]	80.5 [61.3–140.0]	
Unilateral procedure, *n* (%)	5 (6.9)	
Renal denervation system		
Covidien OneSHOT™ (RF), *n* (%)	2 (2.8)	
Paradise (US), *n* (%)	14 (19.4)	
St. Jude EnligHTN (RF), *n* (%)	23 (31.9)	
Symplicity Flex™ (RF), *n* (%)	10 (13.9)	
Symplicity Spyral™ (RF), *n* (%)	19 (26.4)	
Vessix (RF), *n* (%)	4 (5.6)	
Number of ablations	LRA	RRA
Covidien OneSHOT™ (RF), median [25th–75th percentile]	1.0 [1.0–1.0]	1.0 [1.0–1.0]
Paradise (US), median [25th–75th percentile]	3.0 [2.0–3.0]	3.0 [2.0–3.0]
St. Jude EnligHTN (RF), median [25th–75th percentile]	8.0 [8.0–10.3]	8.0 [8.0–9.0]
Symplicity Flex™ (RF), median [25th–75th percentile]	4.5 [4.0–5.8]	5.0 [5.0–6.0]
Symplicity Spyral™ (RF), median [25th–75th percentile]	12.0 [10.0–15.5]	9.0 [6.0–12.0]
Vessix (RF), median [25th–75th percentile]	4.0 [3.3–5.0]	4.5 [0.8–8.0]

*US* ultrasound, *RF* radiofrequency, *LRA* Left Renal Artery, *RRA* Right Renal Artery

^*^Unilateral procedure due to anatomical difficulties on the untreated side

### Efficacy outcomes

**Fig. 2:**
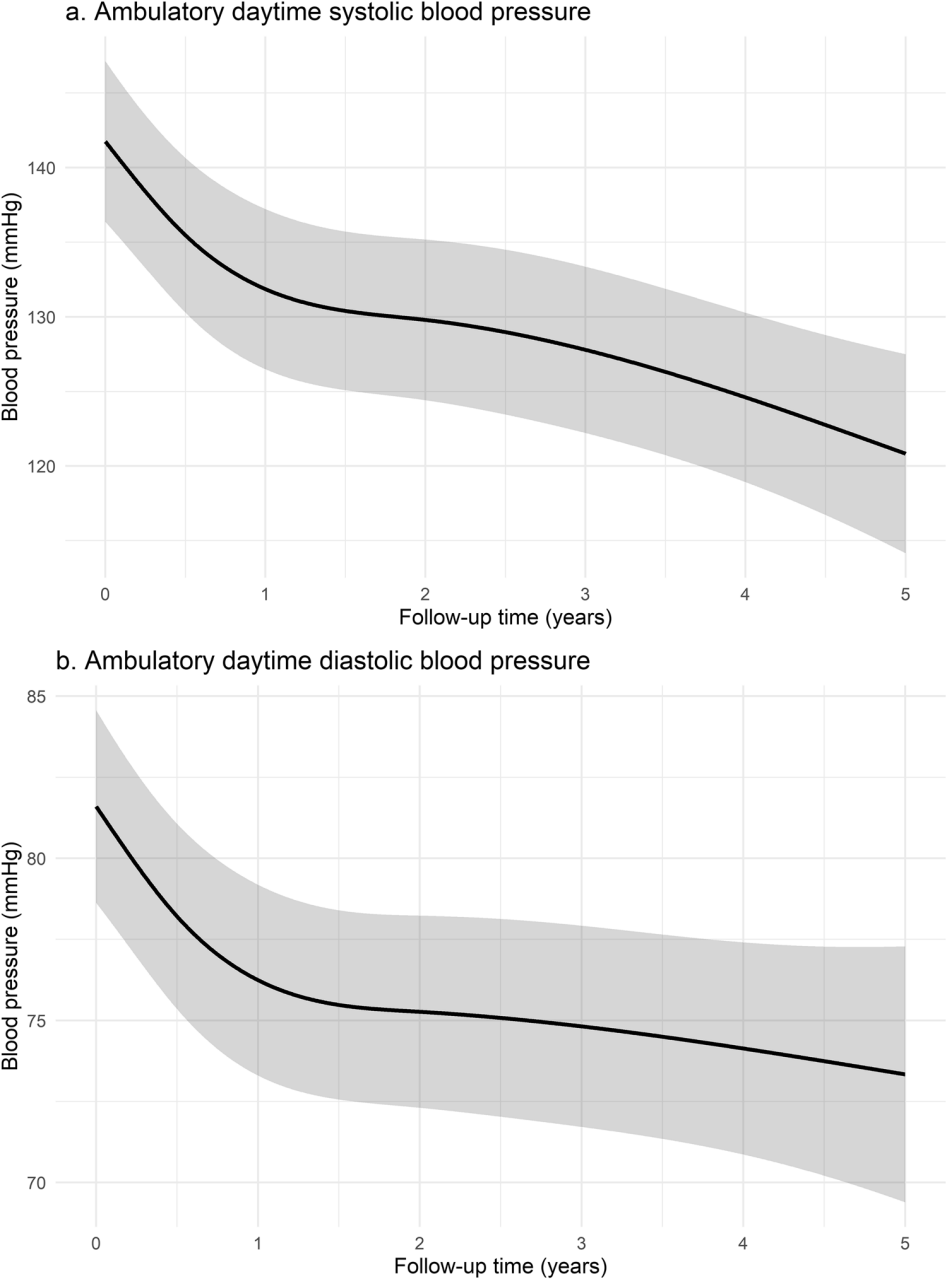
Model effect plot for changes in ambulatory daytime **a** systolic and **b** diastolic blood pressure over time

Both ambulatory daytime SBP and DBP showed a sustained decrease over time after correction for sex, age, and the number of prescribed DDDs (*p* < 0.0001). Estimated ambulatory daytime SBP five years after RDN as derived from the mixed model was 120.8 (95% CI 114.2–127.5) mmHg versus 73.3 (95% CI 69.4–77.3) mmHg for DBP. This implies a modeled reduction of -20.9 mmHg for ambulatory daytime SBP and − 8.3 mmHg for DBP as compared to baseline (Fig. 2). Similar sustained significant decreases over time were observed for all other ambulatory BP measurements (ambulatory mean 24-h SBP and DBP, ambulatory nighttime SBP and DBP; *p* for all < 0.0001; Supplemental Fig. 1, 2). The change in ambulatory daytime SBP over time post-RDN was consistent among predefined subgroups of age (*p* = 0.18), ethnicity (*p* = 0.18), obesity (*p* = 0.24), diabetes (*p* = 0.52), RDN device (*p* = 0.19), and ISH (*p* = 0.82), although a significant interaction between sex and time (*p* = 0.03) was observed (Supplemental Fig. 3).

**Fig. 3:**
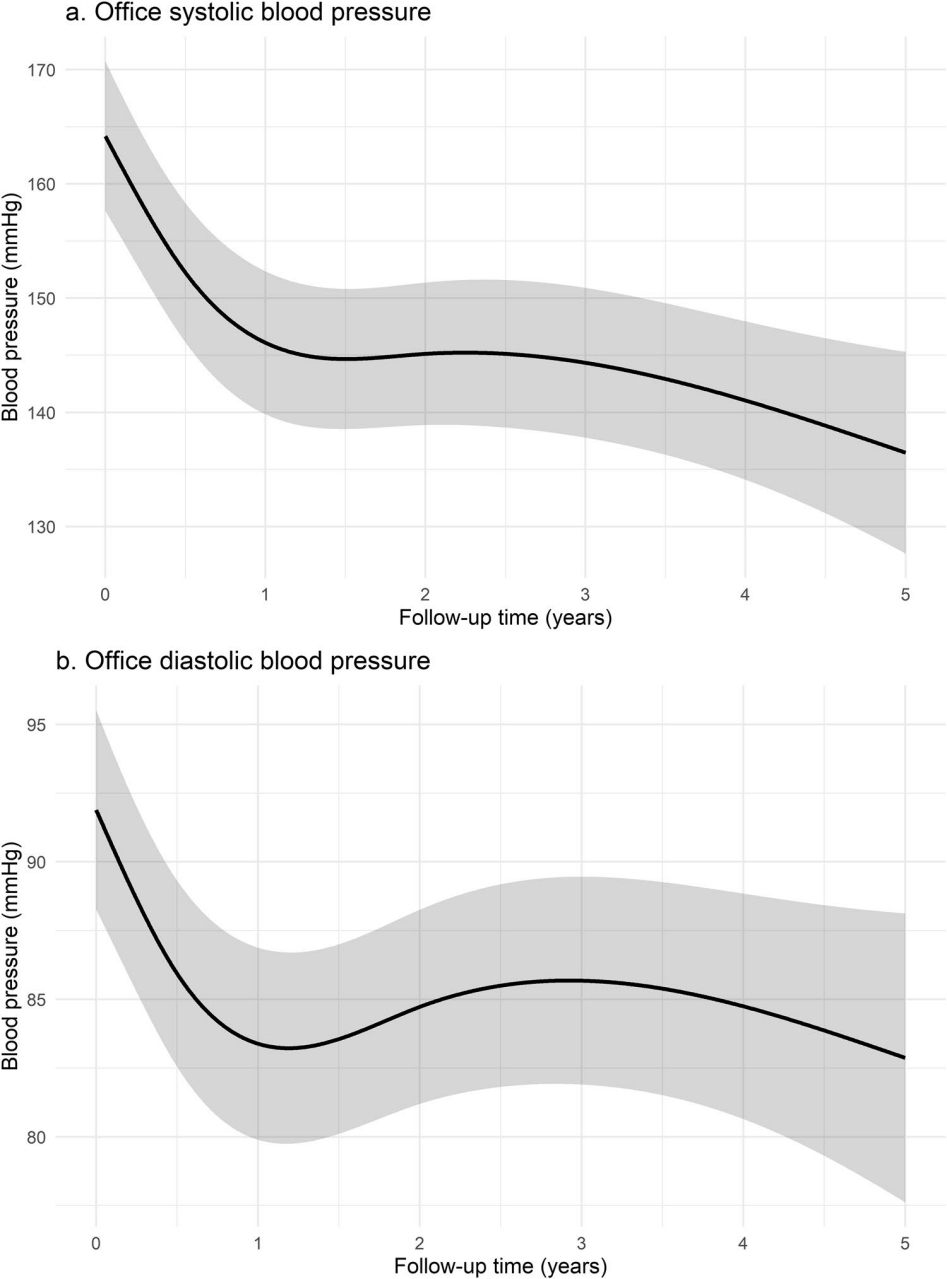
Model effect plot for changes in office **a** systolic and **b** diastolic blood pressure over time

Similar reductions were observed in office SBP (*p* < 0.001) and DBP (*p* < 0.001) over time. At five years post-RDN, estimated office SBP was 136.5 (95% CI 127.7–145.3) mmHg and estimated DBP was 82.9 (95% CI 77.6–88.1) mmHg. The latter implies a modeled reduction of − 27.7/− 9.0 mmHg for office BP as compared to baseline (Fig. 3). Throughout follow-up, a temporary decline in office heart rate was observed (*p* = 0.003; Supplemental Fig. 4).

**Fig. 4:**
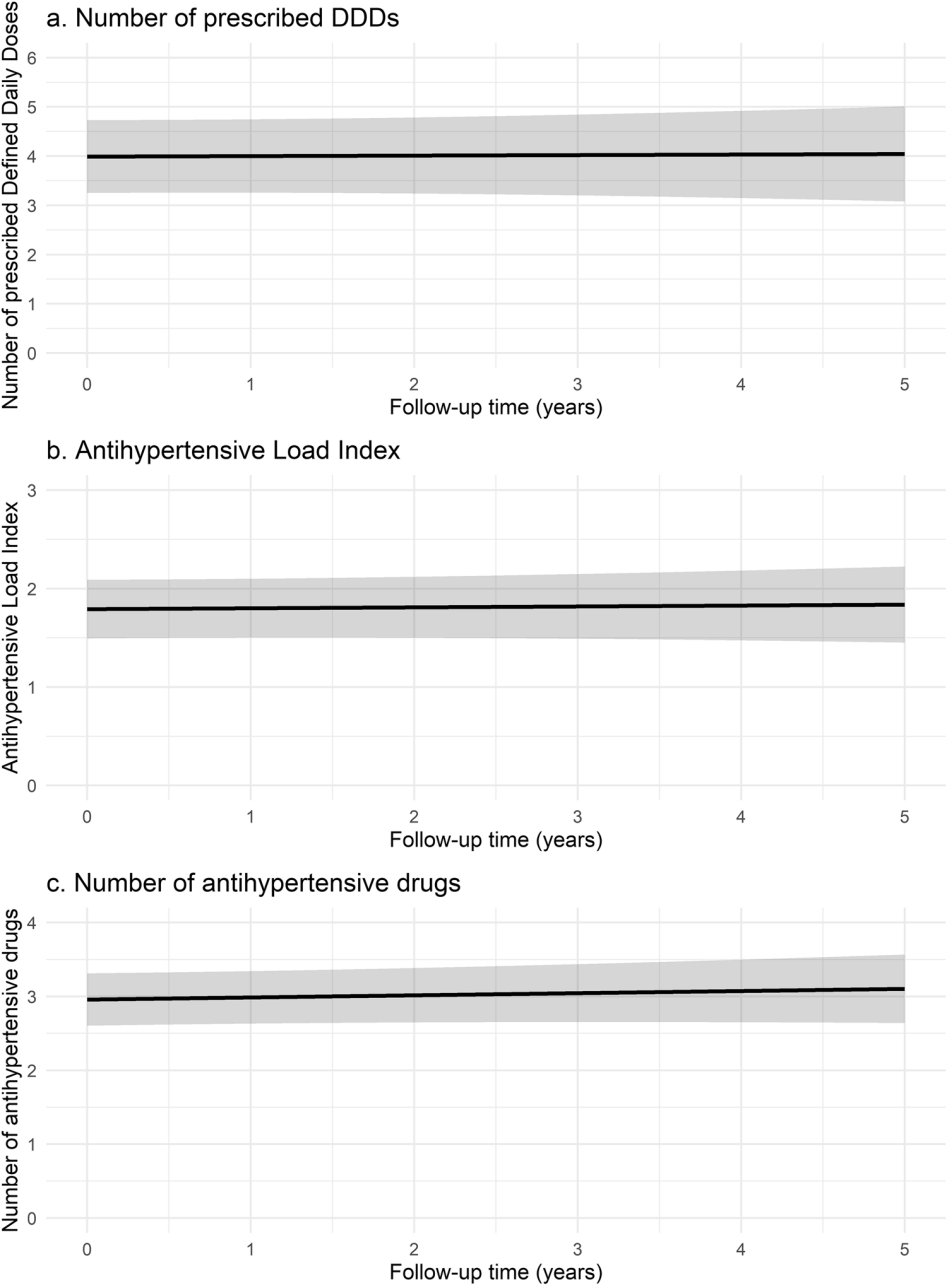
Model effect plot for changes in **a** Defined Daily Doses (DDD), **b** Antihypertensive Load Index, and **c** number of prescribed antihypertensive drugs over time

### Use of antihypertensive medication

The estimated number of antihypertensive drug DDDs five years after RDN was 4.0 (95% CI 3.1–5.0), and there was no modeled change as compared to baseline (0.01 increase per year; 95% CI − 0.12 to 0.14; *p* = 0.87). Likewise, there were no modeled changes in AHLI (0.01 increase per year; 95% CI − 0.04 to 0.06; *p* = 0.74) and the estimated number of antihypertensive drugs per patient (0.03 increase per year; 95% CI − 0.04 to 0.09; *p* = 0.39) during follow-up (Fig. 4).

### Safety outcomes

Three patients (4.2%) suffered from periprocedural complications. One patient had a retroperitoneal hematoma causing hypotension, which was discovered shortly after the procedure. Repeat angiography did not reveal any signs of active bleeding. The bleeding was managed successfully by transfusion of one packed cell and fluid suppletion. The patient was discharged in good condition after four days and repeat renal artery MRA at six and twelve months showed no residual renal artery damage. In the second patient, treatment with the EnligHTN system resulted in a dissection of the right renal artery which was resolved by balloon dilatation. Repeat CTA of the renal artery at one and six months showed no signs of luminal obstruction. In one patient, hypotension occurred in the hours after the procedure with no clinical signs of bleeding complications. BP normalized after discontinuation of all antihypertensive drugs and fluid suppletion and the patient was discharged in a good condition after four days. Uncomplicated renal artery stenting was performed three months post-RDN in one patient who retrospectively showed signs of fibromuscular disease on the renal angiogram. One patient died six weeks post-procedure, most likely due to a cardiac arrhythmia that seemed unrelated to the procedure itself. One patient (74 y/old) with diabetes developed right renal atrophy five years after the RDN procedure, which led to a decrease in eGFR from 80 to 50 ml/min/1.73m^2^ after which renal function and BP remained stable. None of the procedure-related events resulted in long-term morbidity. During a five-year follow-up period, the most frequently observed adverse events were stroke (38.6 per 1000 person-years), coronary revascularization (30.9 per 1000 person-years), and hospitalization for hypertensive emergency (19.3 per 1000 person-years; Table 3).

**Table 3:** Occurrence of adverse events

Safety endpoints (patients at risk)	PP (72)	3 M (71)	6 M (71)	1Y (71)	2Y (67)	3Y (55)	4Y (36)	5Y (30)	Cumulative	Incidence rate (cases per 1,000 person-years)
Major vascular complication	1	0	0	0	0	0	0	0	1	3.9
Post-procedural hypotension	1	0	0	0	0	0	0	0	1	3.9
Renal atrophy	0	0	0	0	0	0	0	1	1	3.9
Stroke	0	0	1	2	5	2	0	0	10	38.6
Myocardial infarction	0	0	1	1	1	1	0	0	4	15.4
Coronary revascularization	0	1	1	2	1	1	1	1	8	30.9
Hospitalization for hypertensive emergency	0	2	1	2	0	0	0	0	5	19.3
Newly acquired renal artery stenosis and/or repeat renal artery intervention	1	1	0	0	0	0	0	0	2	7.7
Renal failure	0	0	0	0	0	0	1	1	2	7.7
All-cause mortality	0	1	0	0	2	0	0	0	3	11.6
Cardiovascular mortality	0	1	0	0	2	0	0	0	3	11.6

*PP* periprocedural, *M* months, *Y* years. Total number of observed person-years = 259

**Fig. 5:**
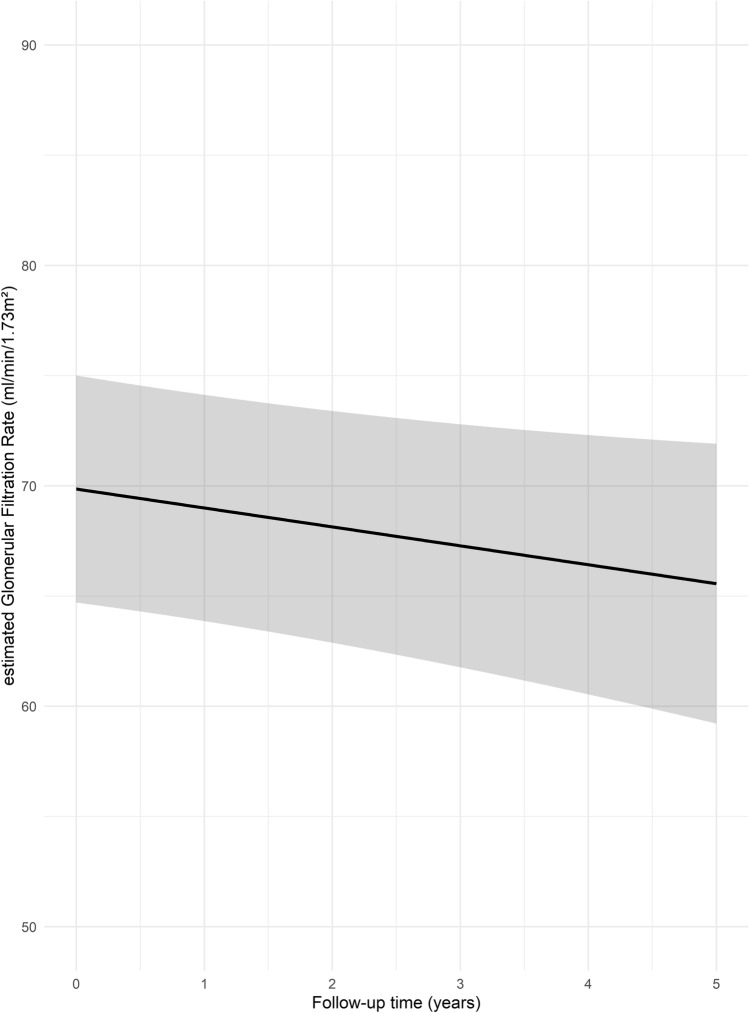
Model effect plot for changes in renal function (estimated glomerular filtration rate; eGFR) over time

Estimated renal function as measured by eGFR was 65.6 (95% CI 59.2–71.9) ml/min/1.73 m^2^ five years after RDN, which implies a modeled annual decline of − 0.86 (95% CI − 1.72 to 0.00; *p* = 0.05) ml/min/1.73 m^2^ (Fig. 5).

## Discussion

To the best of our knowledge, the present study is the first to assess the long-term safety and efficacy of RDN as measured using 24-h ambulatory BP measurements corrected for changes in quantitative drug burden in patients with (therapy resistant) hypertension up until five years post-RDN. We were able to demonstrate a significant and sustained decrease in BP up until five years after correction for age, sex, and antihypertensive drug use throughout follow-up.

With an estimated decrease of − 20.9 mmHg in ambulatory daytime SBP and − 27.7 mmHg in office SBP five years after RDN, our findings support a durable BP-lowering effect of RDN. With respect to RDN safety, there were three major periprocedural complications, whereas one long-term adverse event was reported. In this particular case, the exact pathophysiological relation between newly developed renal atrophy and the RDN procedure five years earlier remains uncertain. The observed annual decrease in eGFR of − 0.86 ml/min/1.73 m^2^ most likely reflects the natural course of renal function over time in patients with (therapy resistant) hypertension. The magnitude of renal function decline observed in our study was in line with previous observations from a large cohort study demonstrating an annual decrease in eGFR of -0.88 ml/min/1.73 m^2^ in patients with a history of hypertension [20]. Whereas our short-term findings are in line with previous work, the present data support the safety of RDN up to 5 years post-procedure [9, 21–25].

The present study has several distinct features that deserve to be emphasized. At first, by protocolized yearly follow-up visits, we were able to incorporate changes in antihypertensive drug burden over time. The latter was acknowledged as a critical confounder in previous studies and was not considered in the largest RDN registry to date with available long-term follow-up data [15]. As such we were able to conclude that the BP-lowering effect of RDN was durable and not linked to a potential increase in drug burden over time.

Second, BP was monitored according to standardized office readings along with the parallel use of standardized 24-h ambulatory BP measurement, which is still considered the gold standard for assessing efficacy in device-based hypertension treatment. The scheduled visits with fixed time intervals allowed for more complete follow-up data as compared to previous literature. The largest study to date reported a CCI of only 47.5% for ambulatory BP and 58.7% for office BP at three years, while systematically higher CCIs of 85.3% for ambulatory BP and 84.3% for office BP were observed in the current study at three years [15]. These findings were consistent up until five years with a CCI of 67.3% for ambulatory BP and 66.4% for office BP. The latter strengthens the validity of the conclusions derived from this study, as the risk of bias related to non-random reporting of BP outcomes reduces with an increase in completeness of follow-up [15].

To account for intra-participant correlation between repeated measurements over time, linear mixed-effects models were used for the analyses of all BP measurements, drug use, and renal function over time. Thereby, all data on outcome measurements gathered during follow-up were used in the efficacy endpoint analysis. As a result, no follow-up data were discarded, loss of statistical power was minimized, and no multiple testing issues arose.

The differences in study design and lack of data on long-term drug burden in most previous studies hamper the comparison of our study to previous work. Previous studies with follow-up data up to 4 years reported decreases in ambulatory SBP ranging from − 8.0 to − 11.0 mmHg along with decreases in office SBP varying between − 7.0 and − 32.0 mmHg, which is in line with the findings of the present 5-year follow-up data [15, 24, 26, 27]. Moreover, our findings were consistent with the 3-year results of the SPYRAL HTN-ON MED pilot trial, reporting a reduction in ambulatory daytime SBP of − 10.2 mmHg post-RDN as compared to sham control in the absence of a between-group difference in antihypertensive drug burden [13].

We found a consistent treatment effect over time in several specific subgroups, including ISH and age. However, a significant interaction between sex and BP over time was observed, which is a finding that cannot readily be explained. Previous RDN trials demonstrated a large heterogeneity in the treatment effect of RDN and were not able to identify consistent predictors of effect, including clinical parameters as well as biomarkers, such as plasma renin activity [8, 9, 11, 23, 28–30]. Future and larger studies are needed to confirm the robustness of these our post hoc findings with respect to sex and identify other predictors of response.

Finally, in the present study, RDN was performed with six different devices, each with their inherent characteristics regarding number of ablations, lesion application location (either proximal or distal), and type of energy delivered (US, monopolar RF energy, bipolar RF energy). Thus far, studies assessing the efficacy of different RDN technologies all showed comparable effect sizes and responder rates [11, 23, 28, 31]. While not being designed to compare individual devices, the present study showed no difference between any of the different technologies. Furthermore, most patients included in the present study were treated before the presentation of the results of the SPYRAL HTN-ON MED and HTN-OFF MED trials in which treatment focus shifted toward a more distal renal artery bed. The sample size of the present study precludes any statements on potential superiority of different treatment strategies or devices.

### Limitations

This study has several limitations. First, this was a registry-based single-center study without a control or sham comparator arm. Therefore, our results rather reflect the real-world use of RDN, performed with different devices, as proposed over the years by RDN working group consensus statements [32, 33]. Inherent to its design, our study included a less clearly defined and more heterogeneous hypertensive patient population. However, the lack of heterogeneity of the treatment effect in all studies thus far among different types of patients might mitigate this potential limitation. Second, our sample size precluded any statements on rare adverse events. Third, information on technical success of RDN was not available due to the absence of periprocedural markers for success. Furthermore, a small proportion of the patients in our study had a unilateral procedure due to anatomical difficulties (Table 2). Finally, the current study lacked drug adherence testing.

### Conclusion

RDN significantly reduced BP up to at least five years post-procedure in the absence of an intensification of antihypertensive drug regimen. Moreover, RDN appeared safe with no major procedure-related late adverse events. Considering the results of this study RDN is a promising adjunctive therapy for patients with (therapy resistant) hypertension.

## Supplementary Information

Below is the link to the electronic supplementary material.

Supplementary file1 (PDF 475 kb)
