# Supplementary material for: Long-term follow-up of patients undergoing renal sympathetic denervation
Source: Clin Res Cardiol. 2022 Jul 18;111(11):1256–68. doi: 10.1007/s00392-022-02056-5 (PMC9622524; doi:10.1007/s00392-022-02056-5)

Supplemental material

Supplemental table 1. Ambulatory and office blood pressure measurements and renal function per follow-up moment.

	**Baseline**	**3 months**	**6 months**	**1 year**	**2 years**	**3 years**	**4 years**	**5 years**
**Ambulatory blood pressure**								
Daytime SBP (mmHg)	146.1 ± 17.4 (n=71)	141.3 ± 19.0 (n=58)	136.3 ± 18.1 (n=65)	137.6 ± 19.5 (n=65)	134.6 ± 18.0 (n=60)	131.2 ± 18.1 (n=51)	131.2 ± 22.4 (n=31)	128.5 ± 11.9 (n=26)
Daytime DBP (mmHg)	83.7 ± 12.2 (n=71)	81.9 ± 11.2 (n=58)	77.6 ± 8.9 (n=65)	78.8 ± 11.3 (n=65)	76.0 ± 9.3 (n=60)	74.2 ± 10.0 (n=51)	72.8 ± 10.1 (n=31)	72.2 ± 8.2 (n=26)
Nighttime SBP (mmHg)	134.0 ± 17.7 (n=68)	130.8 ± 20.3 (n=52)	125.0 ± 20.2 (n=65)	126.5 ± 19.0 (n=64)	124.4 ± 17.6 (n=60)	122.2 ± 18.7 (n=50)	124.5 ± 25.1 (n=30)	123.3 ± 15.0 (n=24)
Nighttime DBP (mmHg)	74.8 ± 12.7 (n=68)	73.8 ± 13.0 (n=52)	68.6 ± 10.0 (n=65)	70.2 ± 10.9 (n=64)	68.1 ± 9.4 (n=60)	66.2 ± 9.1 (n=50)	67.4 ± 11.5 (n=30)	66.0 ± 8.3 (n=24)
Mean 24h SBP (mmHg)	143.2 ± 16.4 (n=72)	139.2 ± 19.2 (n=61)	133.2 ± 17.6 (n=66)	134.5 ± 18.6 (n=65)	132.7 ± 17.9 (n=61)	129.7 ± 19.2 (n=51)	129.7 ± 19.2 (n=31)	126.7 ± 11.9 (n=26)
Mean 24h DBP (mmHg)	81.2 ± 11.5 (n=72)	80.0 ± 11.3 (n=61)	75.4 ± 8.8 (n=66)	76.6 ± 10.8 (n=65)	74.3 ± 9.5 (n=61)	72.0 ± 9.4 (n=51)	71.3 ± 9.8 (n=31)	70.3 ± 7.8 (n=26)
**Office blood pressure**								
Office SBP (mmHg)	169.2 ± 21.2 (n=72)	154.5 ± 25.6 (n=66)	155.0 ± 24.4 (n=68)	150.9 ± 24.6 (n=68)	146.0 ± 19.6 (n=61)	147.6 ± 19.0 (n=49)	146.1 ± 20.0 (n=28)	141.5 ± 22.0 (n=26)
Office DBP (mmHg)	93.0 ± 14.1 (n=72)	87.6 ± 12.6 (n=66)	84.4 ± 12.5 (n=68)	83.9 ± 12.6 (n=68)	84.2 ± 12.0 (n=61)	81.5 ± 11.8 (n=49)	81.8 ± 13.7 (n=28)	78.0 ± 13.2 (n=26)
**Renal function**								
eGFR (ml/min/1.73m^2)	71.7 ± 16.0 (n=72)	71.0 ± 16.2 (n=52)	72.2 ± 18.2 (n=65)	70.4 ± 17.6 (n=69)	68.7 ± 15.9 (n=64)	65.1 ± 17.0 (n=54)	60.2 ± 20.6 (n=33)	58.6 ± 20.7 (n=28)

DBP: Diastolic Blood Pressure; eGFR: estimated Glomerular Filtration Rate; SBP: Systolic Blood Pressure. All values are displayed as mean ± standard deviation (number of observations).

Supplemental figure 1. Model effect plot for changes in ambulatory mean 24-hour (a.) systolic and (b.) diastolic blood pressure over time.

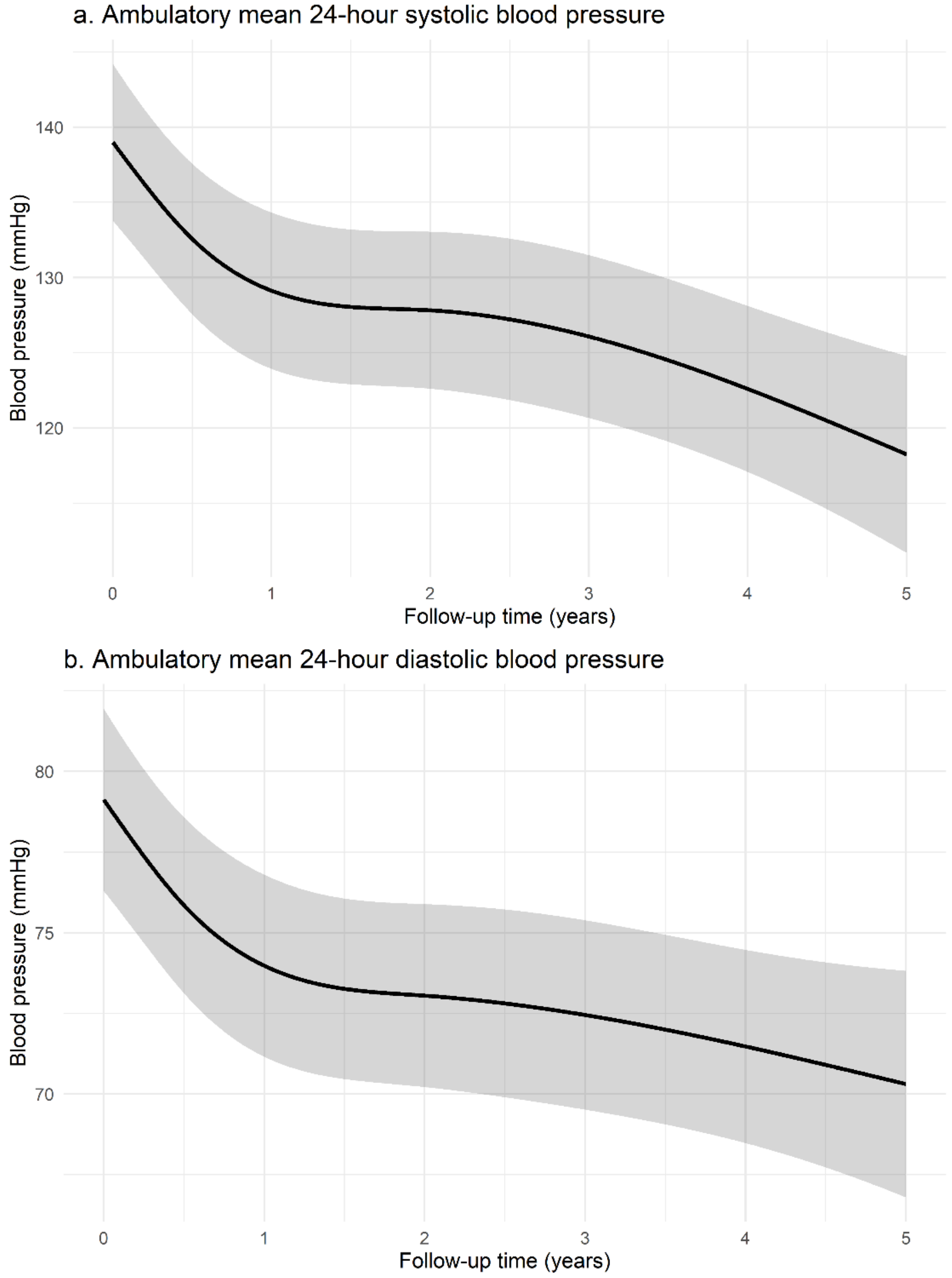

Supplemental figure 2. Model effect plot for changes in ambulatory nighttime (a.) systolic and (b.) diastolic blood pressure over time.

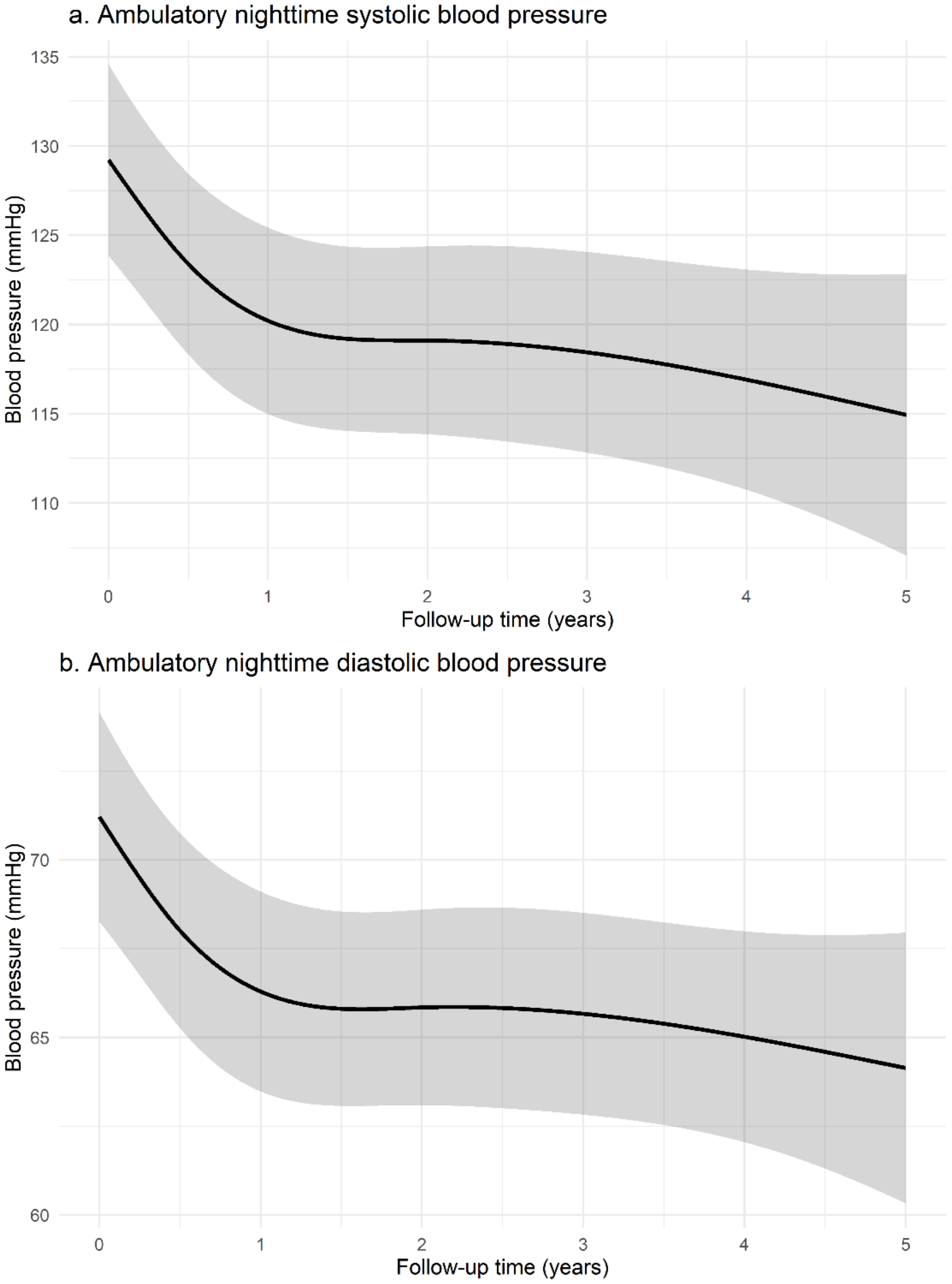

Supplemental figure 3. Model effect plot for changes in daytime ambulatory systolic blood pressure in a subgroup analysis for sex.

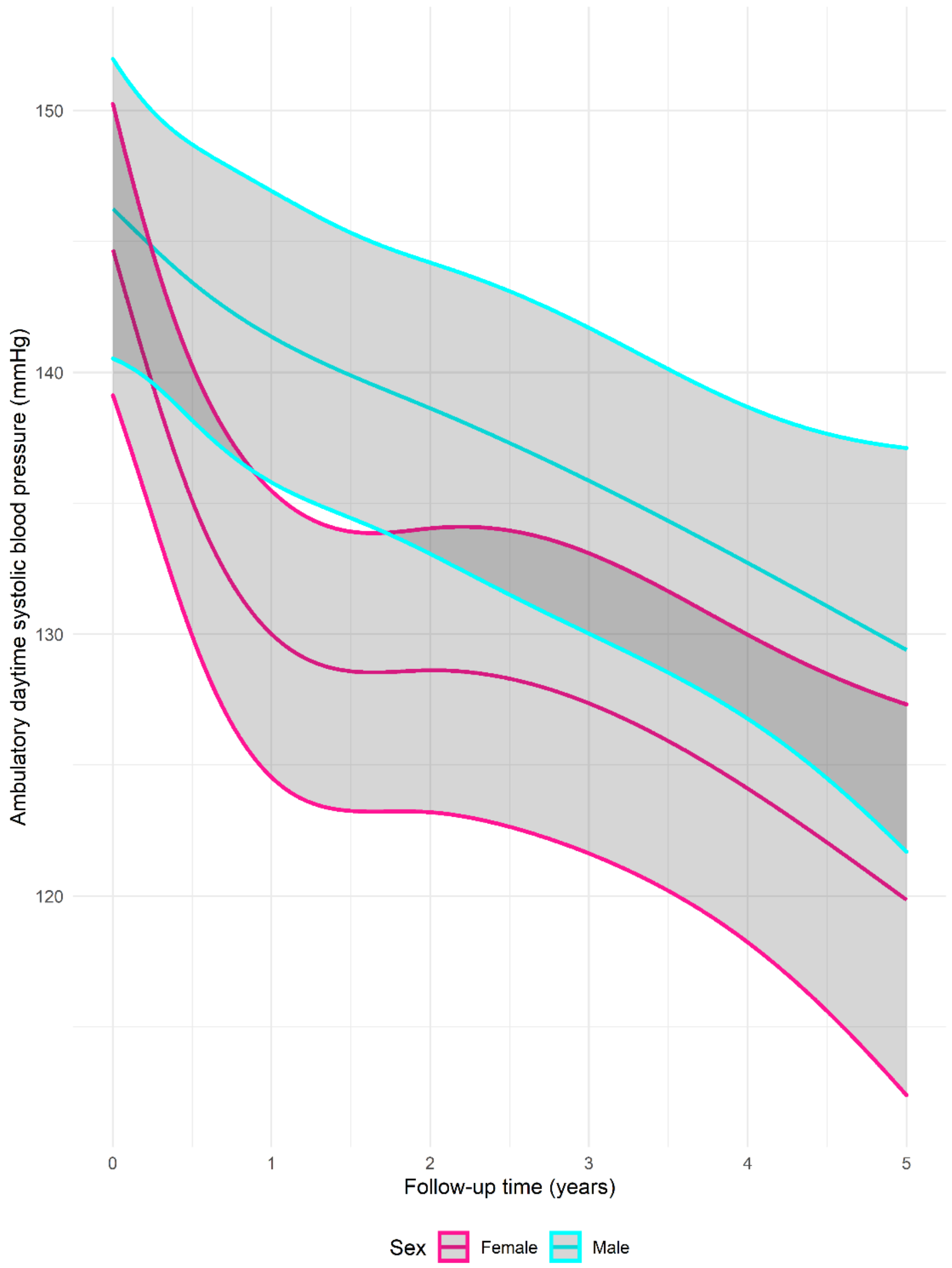

Supplemental figure 4. Model effect plot for changes in office heart rate over time.

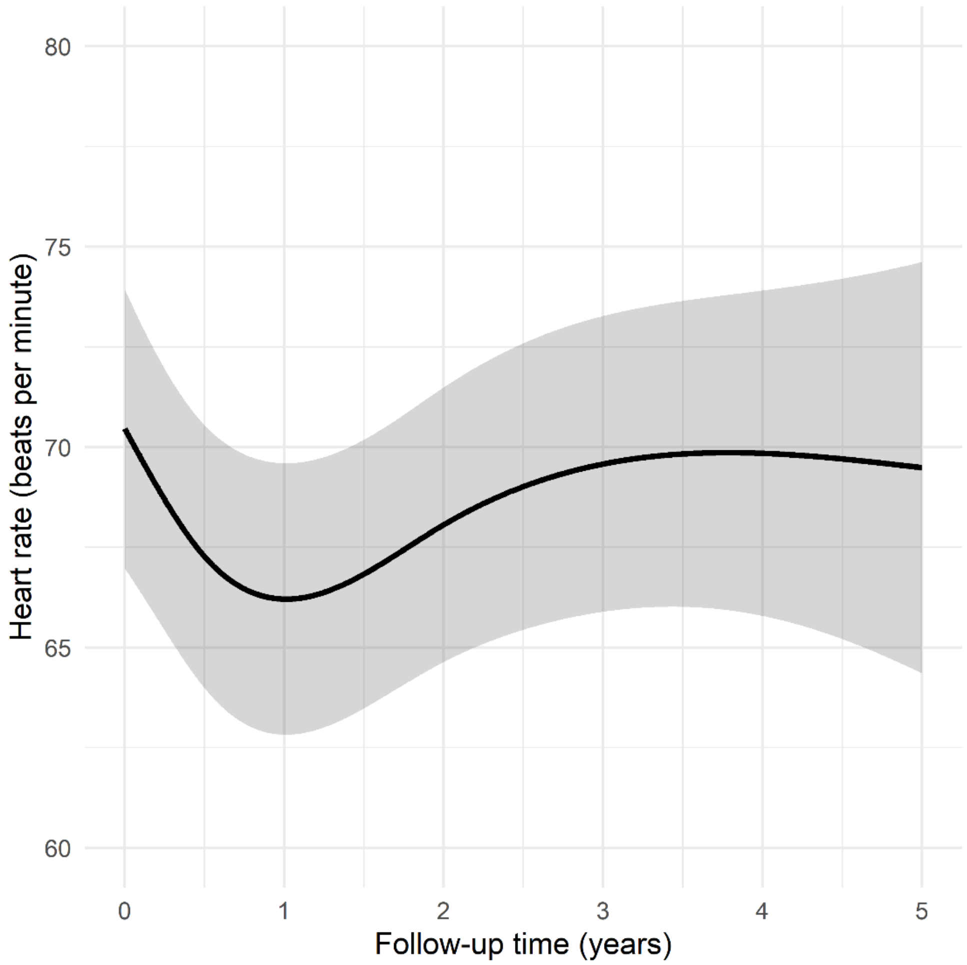

Supplement: Supplementary file 1 — Supplementary file1 (PDF 475 kb) [file 392_2022_2056_MOESM1_ESM.pdf]
